# The proliferation rates of HT-1080 human fibrosarcoma cells can be accelerated or inhibited by weak static and extremely low frequency magnetic fields

**DOI:** 10.3389/fpubh.2025.1535155

**Published:** 2025-06-12

**Authors:** Marek Bajtoš, Nhat Dang, Isabel Lopez de Mingo, Jason Keller, Hakki Gurhan, Ladislav Janoušek, Frank Barnes

**Affiliations:** ^1^Department of Electromagnetics and Biomedical Engineering, Faculty of Electrical Engineering and Information Technology, University of Zilina, Zilina, Slovakia; ^2^Department of Electrical, Computer, and Energy Engineering, College of Engineering and Applied Science, University of Colorado Boulder, Boulder, CO, United States; ^3^Ann and H.J. Smead Department of Aerospace Engineering Sciences, College of Engineering and Applied Science, University of Colorado Boulder, Boulder, CO, United States; ^4^Centro de Tecnología Biomédica (CTB), Universidad Politécnica de Madrid (UPM), Madrid, Spain; ^5^Escuela Técnica Superior de Ingenieros de Telecomunicación (ETSIT), Universidad Politécnica de Madrid (UPM), Madrid, Spain

**Keywords:** weak magnetic fields, J-coupling, fibrosarcoma cells, cell growth, feedback effect, time-delay effect, resonance frequencies

## Abstract

**Introduction:**

Weak static and low-frequency magnetic fields (MFs) have been hypothesized to influence biological systems through mechanisms involving nuclear spin coupling. This study investigates how such fields modulate the proliferation of HT-1080 fibrosarcoma cells.

**Methods:**

HT-1080 cells were exposed in vitro for 4 days to weak MFs with a 10 μT amplitude and frequencies between 12 Hz and 33 Hz, superimposed on a 45 μT static background field. Changes in cell growth, mitochondrial superoxide (O_2_^−^), calcium ion (Ca^2+^) concentrations, and membrane potential were measured.

**Results:**

Results revealed that MFs could either increase or decrease fibrosarcoma cell growth in a frequency- and amplitude-dependent manner. Inversions in growth rates were observed near 16.5 Hz, where a 0.5 Hz shift or amplitude changes as small as 250 nT reversed effects relative to controls. Reversing the static field direction also inverted growth outcomes. Changes in membrane potential, Ca^2+^, and mitochondrial superoxide levels supported a role for bioenergetic modulation.

**Discussion:**

These findings suggest that weak MFs affect cell proliferation through spin-dependent chemical reaction rate changes. The pronounced sensitivity of fibrosarcoma cells compared to normal fibroblasts points to potential therapeutic applications via selective MF-based modulation.

## 1 Introduction

The biological effects of weak magnetic fields (MFs) from power lines, cell phones, electric cars, and other sources have raised concerns as potential causes of increased cellular reactive oxygen species (ROS) that are often associated with cancer, Alzheimer’s, and other age-related diseases (1, 2). Conversely, these fields have also demonstrated potential for treating a variety of diseases (3–5), modulating reactive oxygen species levels and metabolic responses of various cell lines (5–9).

The synergy between electromagnetic (EM) waves and biological systems has been explored in a multitude of studies (10–13). They detail the processes through which extremely low frequency (ELF), weak MF can alter biological systems. For instance, changes evoked by frequencies of 1 Hz, 4.4 Hz, and 16.5 Hz in weak MF intensity range include the activation of fission and regeneration of planarians, inhibition of the growth of Ehrlich ascites carcinoma in mice, stimulation of tumor necrosis factor production by macrophages, reduction in chromatin protection against DNase 1, and enhancement of protein hydrolysis in both *in vivo* and *in vitro* systems (14). Research has shown that weak-combined MFs can influence biological activities such as the division and regeneration of planarians and the retardation of tumor development in animals (15). Certain ranges of ELF MF amplitudes as well as frequencies can induce significant changes to cell viability, such as for umbilical cord blood lymphocytes, but produce no major changes outside of these windows (16, 17). These exposure effects are highly frequency and amplitude-dependent, indicating that specific parameters of MF are more effective at inducing biological changes than others. This behavior accounts for sharp resonance as well as broad biological window effects that act on biological systems.

One proposed mechanism is that ELF MFs increase free radical activity by affecting cell membrane potentials and ion distribution (18). Additionally, research by Timmel and Henbest (19) underscores the ability of weak MFs to influence radical pair mechanisms within biological systems. ELF MFs may increase free radical activity by affecting cell membrane potential and ion distribution, thereby influencing free-radical processes within cells. These radical pair mechanisms are particularly sensitive to MF variations, suggesting that such fields can significantly modulate biochemical reactions by altering spin states and thus affect biological outcomes such as cell growth rates and oxidative stress. This perspective aligns with the observed ability of MFs to alter chemical reaction rates with energy levels well below thermal noise power (20–22).

While numerous studies have investigated the effects of static magnetic fields (SMFs) (23–25), a comprehensive study exploring the interaction of ELF MFs—considering frequency sweep, amplitude sweep and background geomagnetic field—has been lacking. The importance of orientation of static and low-frequency MFs in electromagnetic field (EMF) exposure has been briefly explored, which showed that changing the AC and DC field orientation, relative to the surface the cells grow on, changes the growth rate and production of superoxide. Another study demonstrated lower leaf area values in plants when exposed to artificially reversed geomagnetic fields (26). Additionally, the role of the geomagnetic field has been explored with some studies (27, 28) suggesting the presence of sensory molecules in birds and juvenile Pacific salmon that can detect geomagnetic field direction. However, human *in vitro* studies on geomagnetic sensitivity are very limited (29).

Our hypothesis is that extremely weak MFs can modify biological systems by altering the rate of nuclear spin coupling in certain chemical reactions (e.g., reactive oxygen generation), thereby offering a means to control biochemical reactions in biological systems. The interface between MFs and cells *in vitro* is explored experimentally in terms of AC field amplitude, frequency, and background static DC field magnitude characteristics of the applied MFs. The frequencies and amplitudes of the AC MFs employed in this study ranged from 0 Hz to 33 Hz at 10 μT–30 μT. Since the energy produced by MFs at the mentioned field strengths and frequencies is insufficient to break a biochemical bond, the physical and chemical interactions are likely to involve alteration of chemical reaction rates through the coupling of the applied MFs to magnetic moments in the biological system. These changes, both acceleratory and inhibitory, are predicted to result from alterations in the orientations of proton nuclear spins and their MF coupling to chemically active electrons of the atoms and molecules involved.

This study focuses on HT-1080 fibrosarcoma cells, which is a common and highly recurrent skin cancer that exhibits relative insensitivity to radiation and chemotherapy. It originates from fibroblast cells and has a very high division rate (30). The combined effects of changing both static and ELF MFs were quantified to improve understanding of the interaction mechanisms controlling the growth rates of fibrosarcoma cells. The current study not only investigates human cancer cells in terms of geomagnetic sensitivity but also examines how fibrosarcoma cells respond to pole reversal of the geomagnetic field.

The end parameters examined in this study include growth rate, membrane potential, mitochondrial calcium concentration, and mitochondrial superoxide concentration. Controlling the membrane potential is important as low frequency weak MFs influence vital cellular functions such as homeostasis, signal transduction, proliferation, differentiation, and apoptosis (31). Mitochondrial superoxide (O₂^−^) is a reactive oxygen species generated as a byproduct of the electron transport chain during aerobic respiration in mitochondria. Exposure to ELF EMF can alter the spin states of radical pairs, increasing the rate of O₂^−^ production (32). Furthermore, ELF EMF exposure modulates the activity of electron transport chain complexes, particularly Complex I and Complex III, where O₂^−^ is predominantly produced (33). Mitochondrial calcium (Ca^2+^) is a secondary messenger in response to EMFs, although it is unlikely to be directly affected by the MFs due to the absence of a net magnetic dipole moment in Ca^2+^.

## 2 Results

### 2.1 Theoretical models for predicting weak magnetic effects on biological system

#### 2.1.1 Feedback with a time delay

A circuit model for feedback with a time delay can be used to approximate how changes in the EMFs interacting with an oscillating biological system modify the rate of generation of reactive oxygen species, followed by changes in the concentration or activity of the antioxidant system. It can also be used to model changes in the reverse order where the initial perturbation by the MF is on the antioxidant and followed by a compensating change in the generation of the reactive oxygen species. In either case, the system is represented by two processes separated by a time delay. Using this feedback model (34) enables us to predict the frequencies where we can observe a similar interaction between the biological system and an AC low-frequency field.

For a simple circuit model for a feedback amplifier with time delay (34), the following applies: If the input signal is given by $V_{s}=V_{\text{in}}\cos\mathrm{\omega t}$ and the output signal is given by $V_{o}\cos(\mathrm{\omega t}-\theta )$ , where θ=ωτ and *ω* is the angular frequency and *τ* is the time delay in the feedback loop, the steady state equation can be rewritten as:


$$A_{f}=\frac{V_{o}(t)}{V_{s}(t)}=\frac{A_{o}}{1-\beta A_{o}\frac{\cos(\mathrm{\omega t}-\theta )}{\cos\mathrm{\omega t}}}=\frac{A_{o}}{1-\beta A_{o}(\cos\theta +\tan\mathrm{\omega t}\sin\theta )}$$

The sign of the feedback changes as the phase angle *θ* changes. The term tanωtsinθ varies from zero to ± ∞ with *ω*t so that the overall gain $A_{f}$ oscillates between zero and $A_{f}=A_{0}/(1-\beta A_{o}\cos\theta )$ . Thus, the response of amplifier system is dependent on the angular frequency ω and time delay *τ*. If we examine the system at times when ωt=nπ , the term $\beta A_{o}(\cos\omega \tau )$ changes sign with frequency and $A_{f}$ will increase or decrease from the value for a system with zero-time delay depending on changes in frequency. When $\beta A_{o}\cos\theta =1$ , the system breaks into oscillation with no externally applied signal. To put it simply, this can be understood as pushing a swing to induce a change in motion. If the input is applied at the top of swing in the swing direction of movement, it speeds up, while if it is against the direction of motion, it slows down. Pushing the swing at intermediate points has smaller or no effect at all on the system response. Consider that these biological systems are inherently oscillatory and has a certain gain value from not being at thermal equilibrium, under this model, the input frequency and strength as well as the timing with respect to cellular process can produce drastic differences with small shift in the input parameters.

Biological systems rely on feedback and repair mechanisms that operate with inherent time delays following external stimuli. These delays allow periodic stimuli, such as applied magnetic fields (MFs), to influence system responses by either amplifying or attenuating them, depending on the stimulus timing. Analogous to electronic amplifiers, feedback loops with time delays can induce oscillatory responses, where altering the frequency of the applied MF may reverse the feedback’s effect, shifting the system’s overall response from amplification to attenuation (35). The frequency-dependent nature of biological responses suggests that specific MF frequencies can differentially modulate cellular and molecular processes. By leveraging an understanding of the time constants associated with various biological systems, targeted MF applications could enable precise modulation of critical processes, such as altering cancer cell growth rates or enhancing immune responses.

#### 2.1.2 Proton-proton coupling and nuclear magnetic resonance

A variation in cell parameters due to resonance between an external low frequency MF and fibrosarcoma cells *in vitro* can be initiated by the coupling of the applied external MF to the internal MF generated by the nuclear spins due to the protons and, therefore, the external low frequency MFs would affect the coupling rate of proton to proton involved in nuclear spins in a particular molecular configuration. This coupling would be strongest at frequencies that correspond to the J value, which is equivalent to the energy separating the energy levels of the two nuclear spin orientations with the spins parallel or antiparallel. In turn, protons in this configuration may be coupled to active electrons and change the average population density with a quantum number that is coupled through the MF to the chemical reaction rates that are controlling the growth rate of fibrosarcoma cells and signaling molecules such as hydrogen peroxide.

The importance of nuclear spins in determining the quantum number of molecules in weak MFs is discussed in various papers (20, 36), and the measured results are consistent with expectations for proton-proton coupling. Nuclear magnetic resonance spectroscopy has shown that line widths on the order of a cycle can occur in biological molecules (37, 38).

**Figure 1:**
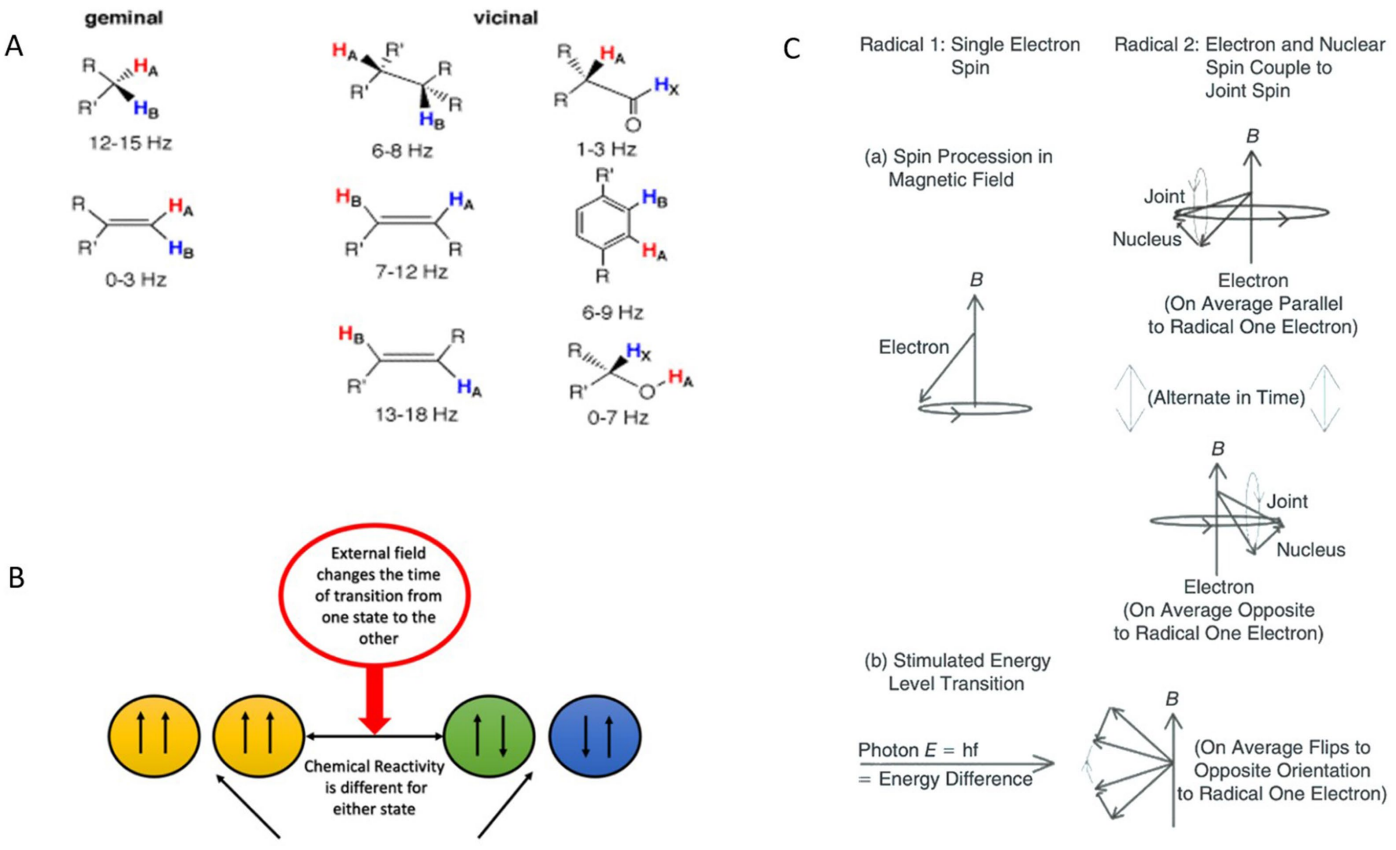
Effects of proton-proton coupling constants, external magnetic fields on spin state transitions and chemical reactivities, and the evolution of spins in a radical pair under different conditions, including precession in a magnetic field and photon-stimulated transitions. **(A)** Examples of proton-proton coupling constants (J) as a function of structure. Reprinted with permission from University of Colorado Boulder, Department of Chemistry and Biochemistry (40). **(B)** Influence of an external MF on the transition times between different spin states and their corresponding chemical reactivities. The external field modulates the transition rate between spin states, resulting in varying chemical reactivities for each state. **(C)** A schematic diagram of evolution of spins of two members of a radical pair, one with only an electron spin and other with both an electron and a nonzero nuclear spin, illustrating changes between relative S and T states under two sets of conditions. (a) Precession of spins in an external MF. (b) Stimulated transition by absorption of photon of energy corresponding to energy difference between levels in one radical. A photon must also carry angular momentum corresponding to the difference between levels. Adapted from (54).

Coupling interactions between protons in large biological molecules have been extensively studied using nuclear magnetic resonance (NMR) spectroscopy, which provides critical insights into molecular structures. The energy level separations corresponding to different proton polarizations are exceedingly small, typically measured in parts per million of the applied frequency or expressed as a coupling constant (J value) in Hertz. The relaxation lifetimes for transitions between these polarization states are generally on the order of seconds, reflecting the slow dynamics of nuclear spin systems. Relevant J values, along with the spatial arrangements of hydrogen atoms, can be referenced in (39), which details NMR frequencies and associated structural configurations. Of particular interest are hydrogen atoms separated diagonally across double-bonded carbon atoms, where the J values fall within the range of 13 to 18 Hz (Figure 1A). These specific interactions and their corresponding frequencies are integral to understanding the effects of external magnetic fields on biological molecules, especially in contexts where nuclear spin dynamics influence chemical reaction rates and cellular processes.

Molecules exhibit J values ranging from 0 Hz to 20 Hz, which typically arise from hydrogen atoms bonded to other atoms and are determined by geminal, vicinal, or long-range couplings (41). Hydrogen protons possess nuclear spins that can align either parallel or antiparallel to each other. These spin interactions can be analyzed using a formalism similar to that applied to radical pairs with correlated electron spins. MFs influence intersystem crossing between singlet and triplet states in radical pairs, leading to notable changes in chemical reaction rates. Specifically, MFs alter the energy levels of the T^+^ and T^−^ states for both electrons and nuclear spins via the Zeeman effect (42). Additionally, time-varying MFs can induce transitions between nuclear spin state energy levels (Figures 1B,C), with these transitions governed by quantum selection rules and the Pauli exclusion principle.

The resulting differences in angular momentum between nuclear spin states translate into distinct chemical activities. For instance, in a hydrogen molecule, configurations with proton spins aligned parallel versus antiparallel exhibit varying chemical reactivity. This principle extends to more complex molecules. Identifying molecules in the biochemical pathways that influence fibrosarcoma cell growth rates presents an intriguing challenge. One candidate is flavin adenine dinucleotide (FAD), a membrane-associated molecule involved in electron transfer from NADH to oxygen, resulting in the formation of O_2_^−^ (43). In particular, the energy levels of trans-olefinic protons include coupling energies between atoms with a J value of 16.5 Hz (39). This frequency shift is detectable in NMR spectra for various molecules, reflecting proton coupling through carbon atoms and involving interactions with elements such as nitrogen and phosphorus. These interactions, and their associated energy shifts, may play a critical role in mediating the effects of MFs on fibrosarcoma cell growth and metabolic processes.

#### 2.1.3 Conformational isomerism

One mechanism for changing chemical reaction rates is through interaction with proteins and enzymes at specific conformational states. Conformational states play a crucial role in the chemical reactivity of both proteins and enzymes. When biological systems interact with ELF EMFs at a frequency equivalent to the J value, corresponding to an energy shift for a change in position or polarization of nuclear spins, only one conformational state of the proteins/enzymes can be influenced. This change in conformational state can alter the reaction rate of a chemical, which is part of a chain of reactions leading to biological changes, as demonstrated by Haspel et al., who highlighted the crucial role of conformational changes in protein functionality and interactions (41). This study measures the combined effects of changes in static fields in the presence of an AC field to improve understanding of the possibility of the Zeeman effect as the interaction mechanism in fibrosarcoma cells.

### 2.2 Magnetic fields affect fibrosarcoma and fibroblast in a frequency-dependent manner

Magnetic field parameters were selected to range from 0 Hz to 33 Hz at a flux density of 10 μT, superimposed on a SMF of 45 μT. These parameters were deliberately chosen as they lie significantly below ionizing frequency thresholds and thermal noise levels, ensuring that the MF strength was insufficient to directly break chemical bonds. Instead, the effects observed at these low frequencies and field strengths likely result from changes in reaction rates mediated by the alignment of proton nuclear spins and their coupling with chemically active electrons.

In the frequency range between 13.5 and 15 Hz, cell density is mostly lowered by the end of the exposure period to within 10% below control. However, at 14.5 Hz, we observed a significant increase in growth at 22.66%, contrary to the overall trend. A transition as small as 0.25 Hz from 14.75 to 14.5 Hz was able to reverse the direction of the EMF exposure effect from inhibitory to accelerating. A similar change was observed with the frequency transition from 15 to 15.5 Hz, modulating the effect of exposure from inert to increasing cell density by 16.40%. These small shifts in EMF frequency correspond to very small energy changes, in the order of 10^−15^ eV, which cannot explain the large modulation in growth observed. Higher frequencies, such as 28 Hz and 30 Hz, predominantly suppressed growth, though the effects were less pronounced. These results suggest a resonance phenomenon at certain low frequencies, driving sharp transitions in growth responses within narrow frequency windows.

Human dermal fibroblasts (HDF) exhibited different frequency-dependent responses compared to fibrosarcoma cells. At 14.5 Hz, fibroblast growth rates increased moderately by 9.87%, while at 15 Hz, a slight decrease of −2.52% was observed. These contrasting outcomes suggest differing cellular mechanisms underlying their responses to MF exposure, possibly involving variations in stress response pathways or cell cycle modulation (Supplementary SI Appendix; Supplementary Figure S1). These findings hold potential therapeutic implications, as the differential response could enable selective targeting of cancer cells while sparing normal cells.

**Figure 2:**
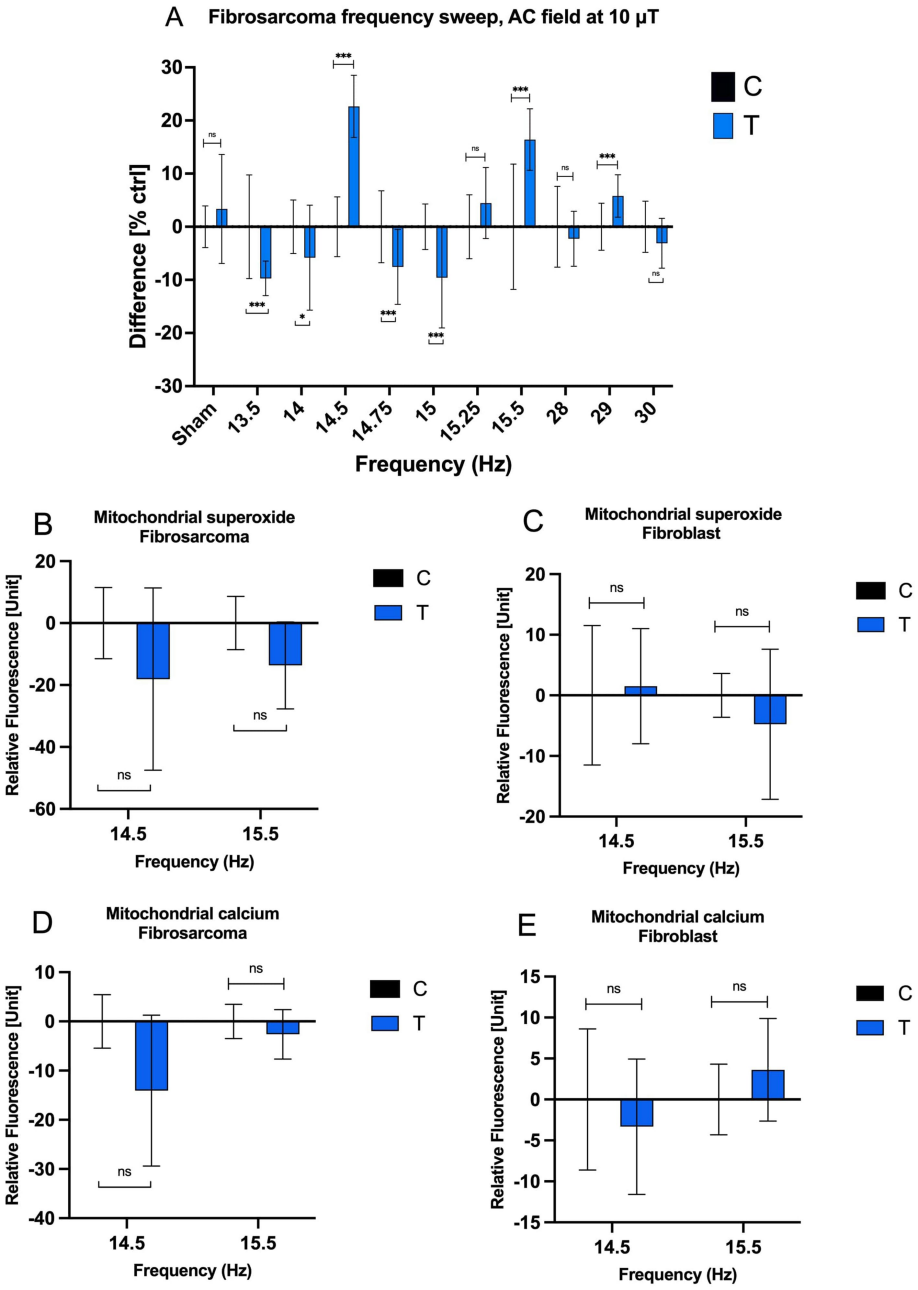
Effects of AC MF frequency on HT-1080 growth rate and mitochondrial chemical balance. **(A)** The cell growth rate of treated and control HT-1080 fibrosarcoma cells as a function of frequency after a four-day exposure. Normalized mean values with +/− SD of treated and untreated samples (*n* = 16 for all experiments, *n* = 14 for Control 15.5 Hz sample, *N* = 1) are presented. AC field = 10 μT (sin-wave), DC field = 45 μT. Mitochondrial superoxide concentration at 14.5 Hz and 15.5 Hz of **(B)** fibrosarcoma and **(C)** fibroblast and mitochondrial calcium concentration at 14.5 Hz and 15.5 Hz of **(D)** fibrosarcoma and **(E)** fibroblast when 10 μT AC field and 45 μT DC field (*n* = 6 well plates by 49 fluorescence samples and *N* = 1) for each bar on the graphs.

To further explore these effects, mitochondrial dynamics were assessed, focusing on changes in mitochondrial calcium and superoxide levels. Responses to 14.5 Hz and 15.5 Hz frequencies were quantified as percentage changes in relative fluorescence units (RFU) from control values (Figures 2B–E). In fibrosarcoma cells, mitochondrial superoxide levels decreased by −18.10% at 14.5 Hz and −13.63% at 15.5 Hz (Figure 2B). Fibroblast cells, in contrast, exhibited a minimal increase in superoxide at 14.5 Hz (+1.67%) and a slight reduction at 15.5 Hz (−4.99%; Figure 2C). Mitochondrial calcium levels in fibrosarcoma cells also declined markedly, with reductions of −14.05% at 14.5 Hz and −2.62% at 15.5 Hz (Figure 2D). Fibroblasts showed a smaller decline at 14.5 Hz (−3.32%) and a slight increase at 15.5 Hz (+3.59%; Figure 2E). These results underscore the heightened sensitivity of fibrosarcoma cells to MFs, particularly at key resonance frequencies, while fibroblasts appear to maintain more stable mitochondrial dynamics.

The sharp growth rate transitions observed at 14.5 Hz, 15 Hz, and 15.5 Hz align with a feedback model involving resonance phenomena (Figure 2). This model incorporates a time delay (*τ*) between 65 ms and 69 ms, accounting for intracellular transit times and the accumulation of signaling molecules such as NO, H_2_O_2_, and Ca^2+^. Molecule diffusion times within a human fibroblast are estimated at ~100 ms for small molecules and ~1 s for larger proteins (44). These diffusion times may be reduced by intracellular electric fields, which can exceed 200 kV/cm, facilitating the rapid transport of ions and signaling molecules. This temporal framework aligns well with the observed resonance frequencies and the rapid shifts in growth rate.

The model proposed by Kandala (45) supports the hypothesis that resonance frequencies, such as those at 14.5 Hz, 15 Hz, and 15.5 Hz, can modulate cellular responses via changes in feedback gain. This is achieved through transitions in the phase and magnitude of the feedback signal, switching between amplification and attenuation. The resonance observed near 30 Hz, likely representing second harmonics, produced weaker effects, potentially due to frequency mismatch with cellular pathways. These findings suggest that the primary biological interactions occur at fundamental resonance frequencies, where the alignment of nuclear spins and magnetic moments can influence reaction kinetics and intracellular signaling.

Sharp resonances, particularly at 14.5 Hz and 15.5 Hz, also correspond to transitions between quantum states of C-H and N-H bonds (T^+^, T^−^, and T^0^ states), supported by the presence of a J-coupling value at 14.5 Hz. These state transitions may drive the observed growth rate changes through mechanisms such as Zeeman shifts in energy levels, influencing ion fluxes and cellular signaling. Additionally, experiments by (46) demonstrate that sinusoidal frequency pulses (2–4 Hz) with MF strengths of 0.02 μT to 0.08 μT can induce ionic current pulses, further supporting the role of resonance-specific interactions.

Taken together, the observed growth rate transitions and mitochondrial dynamics highlight the critical role of resonance frequencies in mediating cellular responses to MFs. The differential sensitivity between fibrosarcoma and fibroblast cells provides a potential avenue for selectively targeting cancer cells while sparing normal tissue. These findings pave the way for further exploration of MF-based therapeutic strategies, leveraging resonance phenomena to modulate cellular growth and signaling pathways.

### 2.3 Magnetic fields affect fibrosarcoma in an amplitude-dependent manner

Building upon Kandala’s results and the frequency sweep data presented in Figure 2, this study further investigated the impact of variations in AC magnetic field (MF) amplitude on fibrosarcoma cell growth rates. The objective was to explore whether small changes in amplitude could account for the differences observed in prior data sets (45). Two AC frequencies, 16.5 Hz and 33 Hz, were selected for analysis, as they demonstrated the most significant effects in Kandala’s frequency sweep. Sinusoidal waveform exposures were applied, with amplitudes ranging from 10 μT to 30 μT, against a 45 μT SMF.

**Figure 3:**
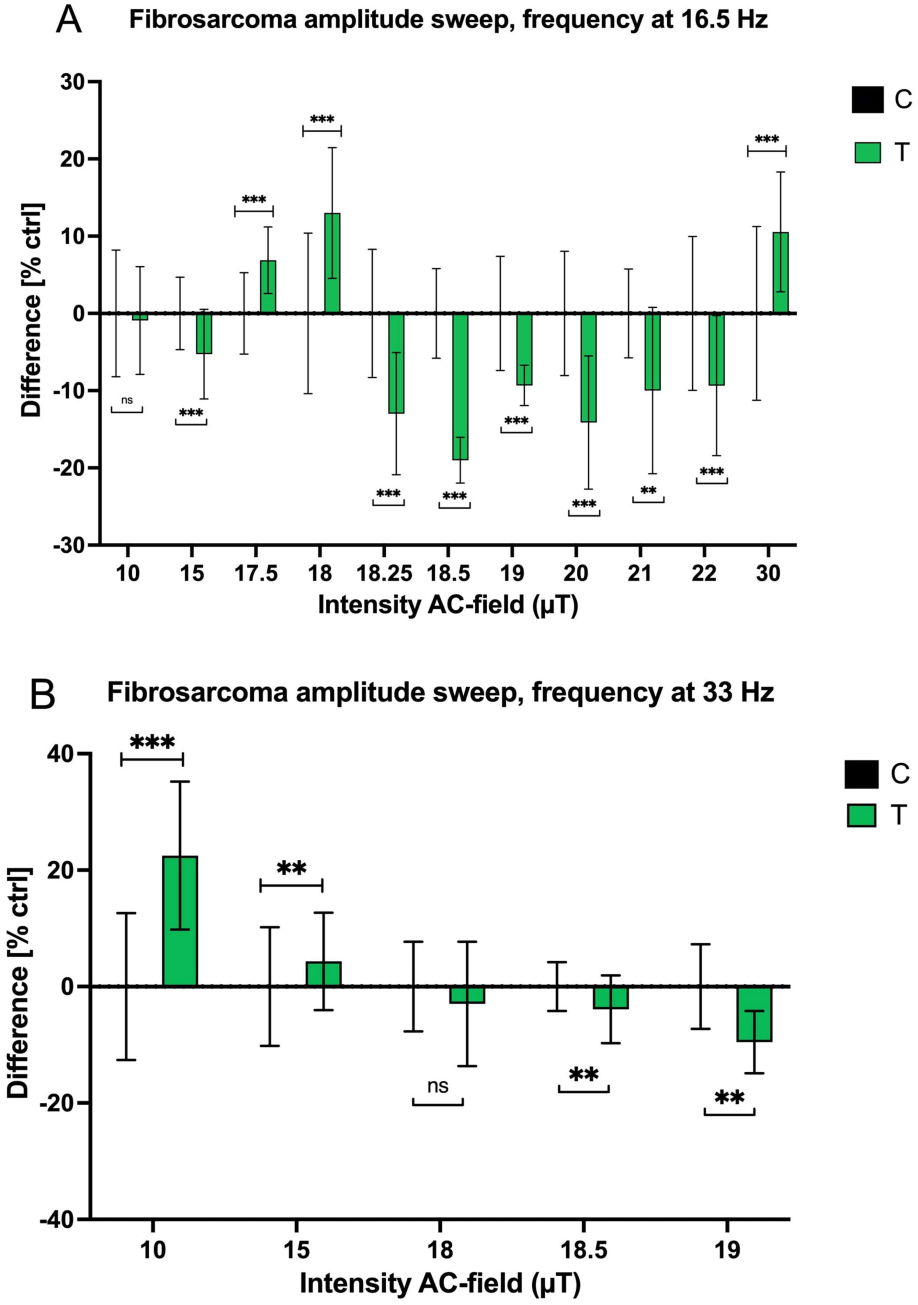
The cell growth rate of treated and control fibrosarcoma cells as a function of amplitude after a four-day exposure. Normalized mean values with +/− SD of treated and untreated samples are presented. **(A)** 16.5 Hz (sinusoidal, *n* = 16 when AC intensity is 10 μT, 15 μT, 18 μT, 18.25 μT, 20 μT, 22 μT, 30 μT, *n* = 8 when AC intensity is 17.5 μT, 18.5 μT, 19 μT, 21 μT; *N* = 1), DC field = 45 μT, **(B)** 33 Hz (sinusoidal, *n* = 16, *N* = 1), DC field = 45 μT.

At 16.5 Hz, growth responses to varying AC amplitudes showed significant variability (Figure 3A). Notably, a peak increase in growth rate of 13.01% was observed at 18 μT, followed by a sharp decline to −19.01% at 18.5 μT. The inhibitory effect persists in the amplitude range of 19 μT to 22 μT. A significant recovery in growth rate (+10.55%) occurred at 30 μT. At 33 Hz, a similar pattern of growth responses emerged (Figure 3B). A significant increase in growth rate (+22.49%) was observed at 10 μT, but higher amplitudes beyond 18 μT, including 18.5 μT and 19 μT, resulted in increasing inhibitory effects. These findings suggest that HT-1080 fibrosarcoma cells exhibit dynamic and non-linear growth response to AC amplitude, and not just frequency, changes. This trend suggests that, like at 16.5 Hz, the response of fibrosarcoma cells to 33 Hz AC fields is strongly amplitude-dependent, with marked shifts between stimulation and inhibition.

A consistent feature across both frequencies was the inflection point around 18 μT, where the direction of growth response reversed. With the geomagnetic field typically ranges between 25 μT and 65 μT, the part of the amplitudes studied here is approximately around the 20 μT envelope from the average geomagnetic magnitude of 45 μT. This deviation from the geomagnetic norm appears to induce cellular stress responses, leading to reduced growth rates. However, the frequency-specific nature of these effects is evident: the inflection at 16.5 Hz is sharper and more abrupt, while the transition at 33 Hz is more gradual.

The resonance observed at 16.5 Hz aligns with nuclear magnetic resonance (NMR) phenomena reported for organic molecules in the range of 4.5 Hz to 17 Hz (39, 47). Notably, the 16.5 Hz resonance appears to influence the growth rates of fibrosarcoma cells *in vitro* (45). Importantly, the resonant frequency remained constant despite minor variations in the SMF of several 10s of μT, suggesting a mechanistic link to nuclear spin transitions. Specifically, these transitions may involve a shift from a nuclear singlet (S) state to a triplet (T^0^) state, followed by electron transfer reactions involving O_2_^−^ and FAD, or the generation of H_2_O_2_ via superoxide dismutase (SOD). The sharp resonance at 16.5 Hz, characterized by narrow line widths (~1 cycle), indicates prolonged coherence times, allowing sufficient interaction durations for biological effects to manifest. This resonance may amplify subtle intracellular processes, such as proton coupling or ROS modulation, ultimately driving the observed growth rate changes.

### 2.4 Magnetic fields affect fibrosarcoma in a frequency-dependent manner with respect to AC and DC fields strength

**Figure 4:**
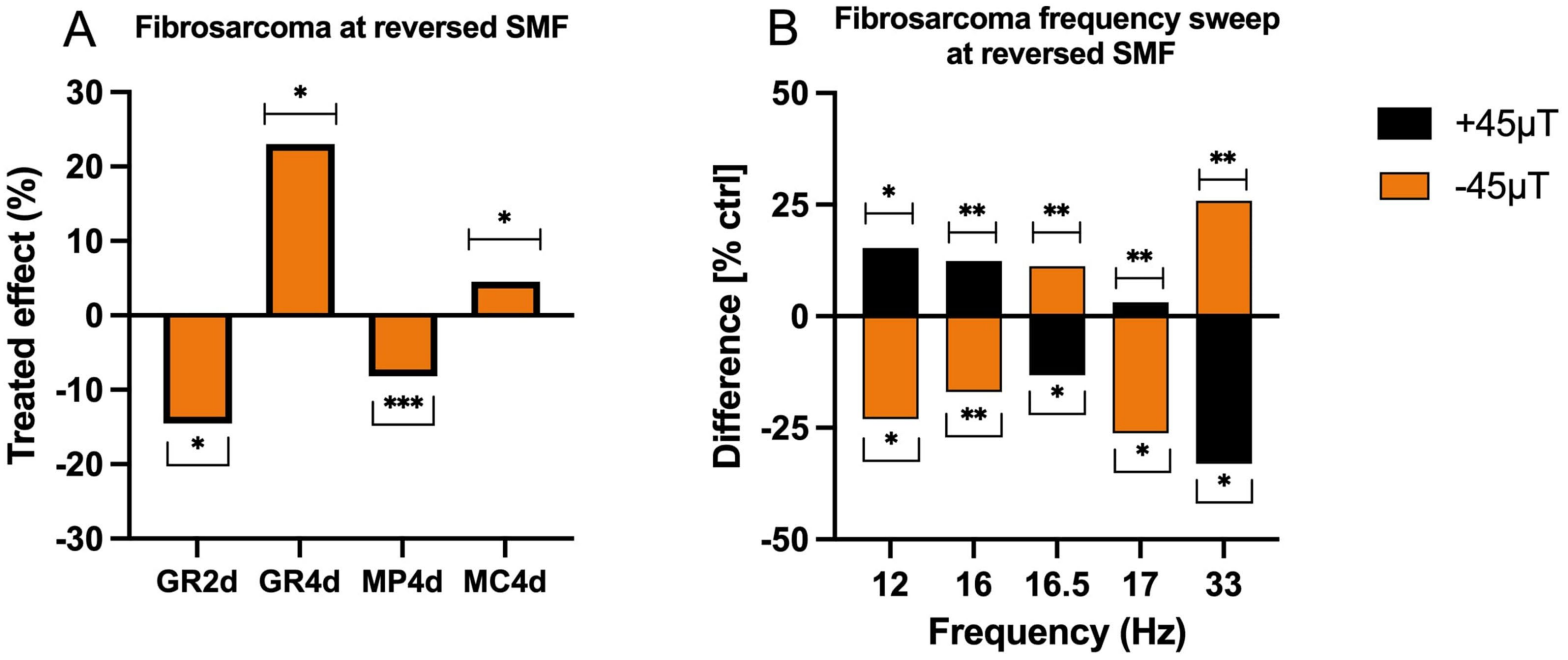
Effects of SMF orientation along the Earth’s surface normal direction (z-direction). **(A)** The percentage difference between the growth rate of a 2-day (GR2d) and 4-day of exposures (GR4d), membrane potential (MP4d) and mitochondrial calcium (MC4d) of the treated and control due to the reversal of the direction of the background DC field, for a 4-day exposure. Subtraction of mean values of treated and untreated samples (*n* = 16, *N* = 1) is divided by mean value of untreated samples to obtain the treated effect [%]. **(B)** The percentage difference between the growth rate of the treated and control as a function of different directions of the DC field of the treated with AC field superimposed. Partially adapted from (45).

The positive geomagnetic field (GMF) direction is defined as the magnetic field lines oriented from the magnetic north pole to the magnetic South Pole, while the negative direction reverses this orientation, exposing cells to field lines in the opposite z-direction. Previous work by Kandala explored the effects of polarity reversal on growth rate, mitochondrial calcium levels, and membrane potential (45). Similarly, the current study assessed these parameters using a SMF of −45 μT, both in isolation (Figure 4A) and in combination with superimposed AC magnetic fields at selected frequencies (12 Hz, 16 Hz, 16.5 Hz, 17 Hz, and 33 Hz; Figure 4B).

When cells were exposed to a reversed SMF (−45 μT) without superimposed AC fields, a significant inhibitory effect on cell proliferation (−14.52%) was observed after 2 days (Figure 4A). This suggests that the initial reversal of the GMF induces a stress response, impairing growth. Interestingly, after 4 days of exposure, the growth rate exhibited a substantial positive effect (+23.05%), indicating an adaptive or stimulatory response to prolonged exposure. Concurrently, changes in cellular parameters were noted: a decrease in membrane potential (−8.19%) suggested disruptions in ionic balance and membrane integrity, while a slight increase in mitochondrial calcium concentration (+4.54%) pointed to modifications in intracellular calcium signaling pathways.

These findings align with Kandala’s hypothesis that fibrosarcoma cells exhibit a dipole moment, possibly associated with nuclear spin orientation. The interaction between a molecule’s magnetic dipole moment and an external magnetic field (MF) can alter chemical reactivity by shifting energy states according to the relationship ΔE=−gmB , where g is the g-factor, m is the internal magnetic moment, and B is the external MF. Maximum energy occurs when m and B are antiparallel. The effect of reversing the GMF is thus consistent with the presence of a permanent dipole moment in fibrosarcoma cells, whose fixed orientation interacts with the applied SMF to alter spin coupling, angular momentum, and reaction rates.

When AC fields were superimposed on the reversed SMF, the frequency of the AC field and the polarity of the SMF significantly influenced growth rates (Figure 4B). At 16.5 Hz and 33 Hz, notable trends emerged based on the background SMF direction. In cases where the background SMF and control fields shared the same direction, the growth rate of the control was higher than that of the treated samples. Conversely, when the background SMF in treated samples was reversed, treated samples exhibited higher growth rates than controls. For other frequencies, such as 12 Hz, 16 Hz, and 17 Hz, distinct polarity-dependent responses were observed. Growth rates were higher when the background DC field was oriented in the positive z-direction and lower when the direction was reversed. At 12 Hz, for instance, the growth rate increased by 15.27% under a + 45 μT DC field and decreased by 23.03% under a − 45 μT DC field. At 33 Hz, the growth rate increased by 25.91% under +45 μT and decreased by 33.08% under −45 μT.

These results highlight the frequency and polarity sensitivity of HT-1080 fibrosarcoma cells to combined MF exposure. The shifts observed with reversed SMF are consistent with the Zeeman effect, where interactions between an external MF and magnetic dipole moments within the cell modulate energy levels. Reversing the DC field alters the resonance frequency, leading to measurable changes in growth rate and intracellular parameters. The observed polarity-dependent effects may be attributed to resonance phenomena, where nuclear spin transitions, influenced by the reversed SMF, affect intracellular processes such as mitochondrial function and calcium signaling. The presence of a static dipole moment in fibrosarcoma cells indicates that SMF directionality can amplify or attenuate these processes, with profound implications for cellular growth and metabolism.

### 2.5 Magnetic fields affect fibrosarcoma and fibroblast in a time-dependent manner

**Figure 5:**
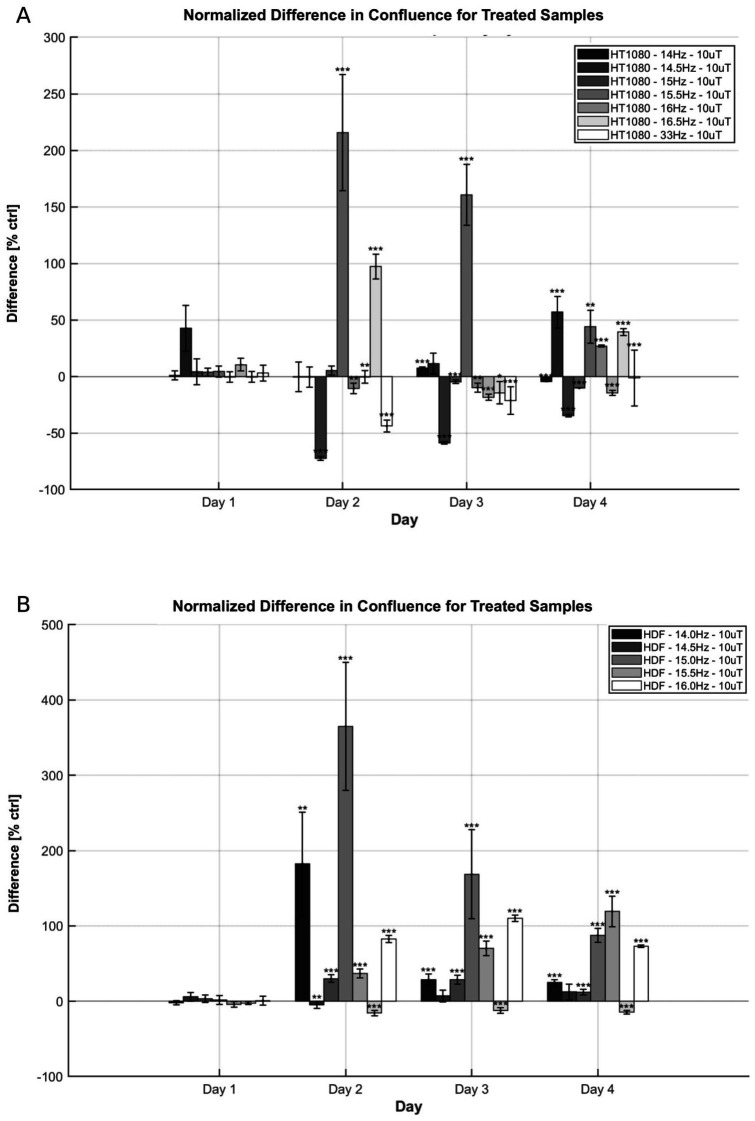
Time-progression effects on confluence of EMF exposure on HT-1080 and HDF. **(A)** Confluence difference for HT-1080 as a function of day and frequency. Normalized means and +/− SD of treated differences (number of wells *n* = 24 or *n* = 60, *N* = 1) are reported. **(B)** Confluence difference for HDF as a function of day and frequency. Normalized means and +/− SD of treated differences (*n* = 60, *N* = 1) are reported. AC field = 10 μT (sin-wave), DC field = 45 μT.

The time-dependent effects of electromagnetic field (EMF) exposure were examined to identify the periods during which modulation of cell growth is most pronounced. Confluence of HT-1080 fibrosarcoma cells and HDF cultured in well-plates was measured daily over a four-day period. All cells were incubated under identical conditions for the first 24 h and exposed to EMF beginning on day 2. For HT-1080 cells, confluence trends during the four-day exposure generally aligned with frequency sweep data previously reported for frequencies with significant effects—namely, 14, 14.5, 15, and 15.5 Hz (Figure 5A).

At 14.5 Hz and 15.5 Hz, confluence increased by the end of the exposure period, reaching 55.90 and 44.12%, respectively. These values surpass corresponding changes in cell count, although they follow similar overall trends, with the final increase at 14.5 Hz being slightly greater than at 15.5 Hz. In contrast, exposures at 14 Hz and 15 Hz showed a decline in confluence by day 4, consistent with the frequency sweep results. Time-resolved analysis revealed additional differences in growth dynamics. Results at 33 Hz exposures on day 4 were also in agreement with trends reported by Kandala; while results at 16.5 Hz have opposite direction at day 4 but similar direction at day 3 (45). As with other exposure conditions, changes in confluence did not directly correspond in magnitude to the smaller shifts observed in total cell count.

At 15.5 Hz, the maximum increase in confluence occurred on day 2, followed by a gradual decline over the next 2 days. In contrast, exposure to 14.5 Hz led to an initial suppression of confluence on day 2, followed by a steady increase through day 4. Although both frequencies ultimately resulted in elevated cell counts by the end of the experiment, their distinct temporal profiles suggest differing resonance interactions with the EMF. Similar divergences were observed between the 14 Hz and 15 Hz conditions. Cells exposed to 14 Hz exhibited an oscillatory pattern, with a confluence increase on day 3 followed by a decline on day 4, whereas 15 Hz exposure produced a continuous decrease over the four-day period.

HDF cells were also exposed across the 14–16 Hz range for comparison (Figure 5B). A general increase in growth rate was observed across all tested frequencies. At 14 Hz and 15 Hz, the confluence peaked on day 2 but declined to a lower value by day 4. In contrast, 15.5 Hz exposure produced a progressively increasing confluence relative to control over the full period, while 14.5 Hz resulted in a relatively stable enhancement from day 2 to 4. These variations in time-dependent responses suggest that EMF-driven resonance in HDF cells may involve multiple time constants and distinct regulatory mechanisms.

## 3 Discussion

### 3.1 Fibrosarcoma can be modulated by weak ELF EMFs

The study by Giuliani and Soffritti significantly advanced the understanding of how extremely low-frequency electromagnetic fields (ELF EMFs) interact with biological tissues, extending beyond the traditional view that such interactions are solely thermal in nature. Their research demonstrated that ELF EMFs can influence crucial cellular functions, such as ion channel activity, cellular differentiation, and stress responses, indicating that these fields have broader biological impacts than previously understood (48). Ion channels, essential for maintaining electrochemical gradients across cell membranes, can be modulated by ELF EMFs, leading to alterations in cellular excitability and function. This suggests that ELF EMFs, by interacting with cellular structures, could significantly influence processes like cell proliferation, differentiation, and apoptosis.

Building on this, Gurhan et al. explored the effects of static magnetic fields (SMFs) on HT-1080 fibrosarcoma cells, emphasizing the role of both the intensity and orientation of the SMF in shaping cellular responses (49). Their findings indicated that the orientation of the magnetic field relative to the cell culture surface plays a critical role in modulating cellular outcomes. Specifically, when the magnetic field was applied perpendicularly to the cell surface, the growth rate of fibrosarcoma cells increased significantly compared to when the field was applied in parallel. Moreover, the direction of the static field relative to the Earth’s geomagnetic field appears to be an important factor influencing biological outcomes, a notion supported by other studies investigating the effects of magnetic fields on cellular behavior (50). We suggest that there exists a permanent magnetic dipole moment aligned with either gravity or the polarity of the cell. This magnetic dipole can be an artifact of the *in vitro* growing environment: the rigid growing surface significantly influences the cell morphology and cytoskeletal response, and orientation this morphological bias is fixed with respect to gravity. Further experiments are needed to verify these effects to isolate EMF-only impact on cellular growth rate as well as identify factors that induce the dipole response.

In this study, the effects of combined AC and static magnetic fields on fibrosarcoma cell growth were further explored, as shown in Figure 2A. Fibrosarcoma cells were exposed to an AC magnetic field at 10 μT with frequencies ranging from 0 to 30 Hz, superimposed on a static magnetic field of 45 μT, matching the SMF applied to the control sample. Data revealed metabolic changes in fibrosarcoma cells, particularly around 14.5–15 Hz, suggesting that the external magnetic field may interact with nuclear proton spins. This interaction likely alters the coupling rate between nuclear spins, which, in turn, shifts chemical reaction rates by modulating the alignment of these spins in parallel or antiparallel orientations. The peak of this coupling effect occurs at frequencies matching the J-coupling constant, corresponding to the energy gap between spin states. At these frequencies, protons in large biological molecules can be coupled with active electrons, influencing the population densities of quantum numbers related to reaction rates, thereby affecting fibrosarcoma cell growth and signaling molecules such as H_2_O_2_.

A comparison was also made between the effects of these magnetic field exposures on fibrosarcoma cells and normal human fibroblast cells. The observed differences in the growth rates of these cell types likely stem from intrinsic biological differences between cancerous and normal cells. Fibrosarcoma cells, being cancerous, exhibit distinct metabolic behaviors, signaling pathways, and responses to external stimuli compared to normal fibroblasts. Cancer cells, due to their uncontrolled proliferation and higher metabolic activity, may be more responsive to external factors such as electromagnetic fields. This heightened sensitivity could explain the pronounced changes in growth rates observed in fibrosarcoma cells at specific magnetic field frequencies and amplitudes. These findings highlight the potential to use electromagnetic fields to selectively target cancer cells while sparing normal cells. This approach could offer a non-invasive cancer therapy that complements existing treatments, such as radiotherapy and chemotherapy, by sensitizing cancer cells to therapeutic agents and ionizing radiation.

In Figures 3A,B, the growth rate response of fibrosarcoma cells as a function of magnetic field amplitude was explored, particularly focusing on the behavior at 16.5 Hz. Results revealed rapid variations in growth rates after a 4-day exposure at 16.5 Hz, coinciding with the J-coupling between proton spins in large biological molecules and the long relaxation lifetimes of nuclear spins. At 33 Hz, a gradual decline in growth rates was observed as the AC field amplitude increased. These changes in growth rates, particularly at lower frequencies and certain field strengths, are likely the result of alterations in chemical reaction rates induced by changes in proton nuclear spin orientations. This interaction suggests that weak magnetic fields may modulate biological systems by influencing the spin states of nuclei and their interactions with chemically active electrons. The amplitude-dependent responses of fibrosarcoma cells to AC MFs at 16.5 Hz and 33 Hz reveal critical insights into cellular sensitivity to magnetic field parameters. Future studies should further delineate the underlying mechanisms, potentially enabling the development of precision-targeted magnetic field therapies for cancer treatment.

Biological systems inherently involve numerous signaling pathways and amplifying mechanisms, with more than 3,000 signaling proteins contributing to hundreds of distinct cellular pathways. Time delays in biological responses, including those of immune and repair systems, are common (34). The results of this study suggest that the timing and parameters of periodic stimuli, such as magnetic fields, can significantly affect cellular reactions, either amplifying or attenuating biological responses. The frequency, amplitude, and static field strength of the applied electromagnetic fields influence cellular chemistry (as shown in Figures 2–4), potentially altering cell behavior in ways that could be leveraged for therapeutic applications. These findings highlight the potential of using electromagnetic fields to selectively target cancer cells while sparing healthy tissue, offering a non-invasive, cell-sparing approach to cancer treatment. Such applications could complement conventional therapies like chemotherapy and radiotherapy, enhancing their effectiveness and minimizing side effects. Overall, this study contributes to a deeper understanding of how weak electromagnetic fields affect biological systems, with significant implications for both medical safety and the development of novel therapeutic strategies for a variety of diseases.

### 3.2 Acute and long-term effects of EMF exposure

This study investigated the time-dependent effects of EMF exposure at various frequencies in HT-1080 fibrosarcoma cells and HDF. The results demonstrate that EMF exposure elicits frequency- and cell-type-specific temporal responses, suggesting that even when long-term outcomes appear similar, the underlying resonance mechanisms may differ significantly. This distinction is particularly evident in HT-1080 cells exposed to 14, 14.5, 15, and 15.5 Hz. Frequency sweep data indicated that exposures at 14.5 Hz and 15.5 Hz (at 10 μT) enhanced cell density by the final day, while exposures at 14 Hz and 15 Hz produced inhibitory effects (Figure 2A). However, daily confluence measurements (Figure 5A) reveal that the temporal dynamics of these effects differ markedly across frequencies. At 15.5 Hz, the maximal difference between treated and control confluence occurred at day 2, just 24 h after exposure began, followed by a gradual decline. In contrast, the 14.5 Hz exposure resulted in a steady increase in confluence difference over the entire exposure period, reaching its peak on day 4.

Similarly, exposures at 14 Hz and 15 Hz, both associated with growth inhibition at day 4, exhibited different time progression. At 14 Hz, the inhibitory effect peaked at day 2 and then plateaued, while at 15 Hz, the suppression of confluence declined progressively over the subsequent days. These patterns suggest that similar endpoint effects may arise from temporally distinct biological processes. In HDFs, EMF exposure also induced both short-term (within 24 h) and long-term (beyond 72 h) changes in growth, further supporting the presence of multiple temporal response pathways.

Collectively, these findings point to several key conclusions. First, each resonance frequency appears to be associated with a distinct cellular response time constant. Second, frequencies that yield similar acute effects—such as 14 and 15 Hz—may diverge significantly in their long-term impact. This highlights the importance of temporal resolution in evaluating EMF bioeffects. Third, the presence of significant short-term effects in HT-1080 cells, but not in HDFs, suggests the potential for cell-selective EMF applications, including targeted therapies that require only brief exposures.

Further investigation is needed to characterize these time constants across cell types and exposure conditions. Given the inherent complexity and heterogeneity of cellular systems, it is likely that multiple molecular targets with distinct kinetic profiles are involved. Identifying these biological and biochemical targets is a critical next step to understanding the mechanisms of EMF-induced resonance responses.

### 3.3 Future directions

While this study was able to provide initial evidence of interaction between weak EMFs with biological systems, it does not fully describe the relationship between frequency, amplitude, and waveform of the EM stimulation on cellular response. The primary cellular response, which is cell density at the end of exposure, while is helpful as a preliminary indication of change in metabolic activities, is not sufficient to illuminate on the exact point of interaction of EMF. As characteristic of cancer cells, an increase in respiratory rate, aerobic glycolysis, and generation of biomass for proliferation, may not reflect from changes to mitochondrial properties that were measured in this study. Further assessment into respiration indicators, such as glucose intake and lactate secretion, will be required in future studies to identify how weak EMF exposure interacts with the metabolism of cancer cells. The correlation between oxidative stress and antioxidant activity of the cell will also need to be studied to verify whether the electron transport chain and oxidative phosphorylation—where there’s a large movement of charges and radical generation, are affected independently from glycolytic processes.

The study is also currently limited to only two cell types: HT-1080 fibrosarcoma and human dermal fibroblast. Assessment of the effect of EMF exposure on other cancerous and healthy cell lines is needed to validate the results. Cancer varies significantly in metabolic activities, gene expressions, protein contents, and mutation, and EMF might interact distinctly with different cancer cell types. A broad database of both frequencies, amplitudes, and temporal response for various cancers and their healthy counterparts will be helpful in the development of non-invasive therapeutic methods, targeting only the harmful components within the tissue.

## 4 Materials and methods

### 4.1 Experiment and exposure system setup

**Figure 6:**
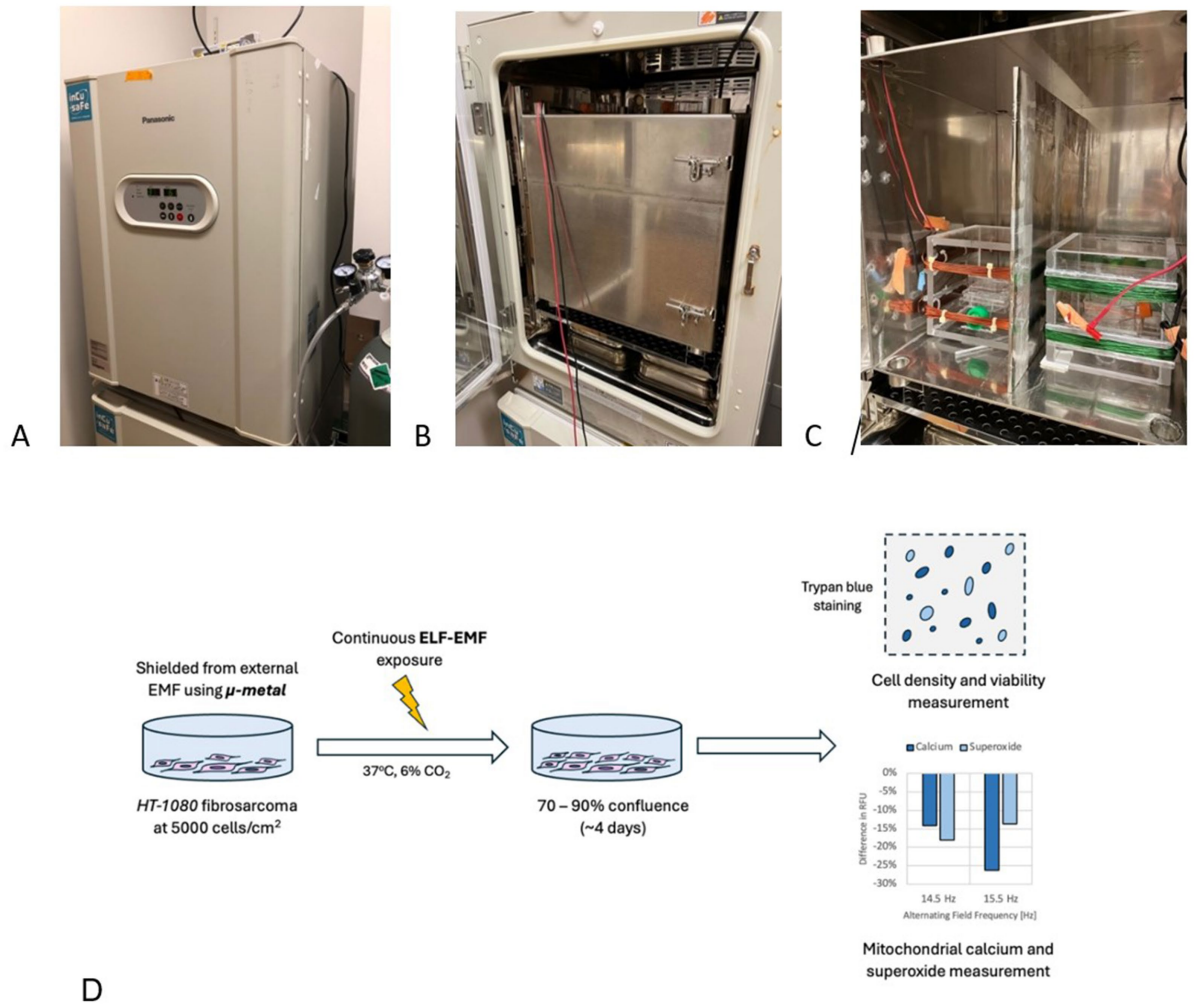
Experimental setup and procedure for EMF experiments with HT-1080 fibrosarcoma. **(A)** Panasonic MCO-18 AC-PE CO2 Incubator in closed configuration, set to 37°C and 6% CO_2_. **(B)** Mu-metal box in closed configuration situated within the incubator to shield electronics emission. **(C)** Opened Mu-metal box shows two coil setups: control (left) and treated (right). A Mu-metal plate is used to shield the two coils from interfering with each other. **(D)** General experiment procedure: HT-1080 fibrosarcoma cells are seeded at 5,000 cells/cm^2^ and are exposed to a selected EMF parameters for 4 days, when cell count results are taken.

The experimental setup comprises three main components: an incubator, a Mu-metal box for static field isolation, and an exposure system (Figures 6A–C). The incubator used is a Panasonic MCO-18 AC-PE CO2 Incubator (Panasonic, Japan) with external dimensions of 62 × 71 × 90 cm. The internal cavity measures 49 × 52.3 × 66.5 cm, resulting in a total internal volume of 170 liters. Within the incubator, a 42-cm cube Mu-metal box is positioned 14.3 cm from the internal floor. This Mu-metal box has a usable volume of 39 cm3, divided into left and right chambers by a 1 cm-thick acrylic divider plate wrapped with 0.05 mm-thick Mu-metal. An exposure system is placed in each chamber to independently control the electromagnetic fields (Supplementary SI Appendix; Supplementary Figure S2). The Mu-metal shielding ensures minimal interference from external magnetic sources, creating a controlled and uniform magnetic environment. This design aligns with the MagShield apparatus described by Vučković et al. (51), which integrates Helmholtz coils within mu-metal enclosures to enhance field uniformity and reproducibility. The system supports simultaneous experimental and control conditions using separate chambers, a critical feature for minimizing confounding variables in biological assays. Such isolation is essential, as variations in geomagnetic and other background magnetic fields can significantly impact experimental outcomes (52).

The incubator maintains a CO_2_ concentration of 6% at 1 atm total gas pressure, supplied directly from an external tank. Humidity control is achieved using deionized (DI) distilled water provided by two trays, each containing 1,400 mL of water. These trays have an air-exposed surface area of 520 cm2 (20 cm × 26 cm) and a depth of 4 cm. The incubator temperature is set to 37°C to simulate physiological conditions.

The exposure system consists of a 4-faced acrylic box with sides measuring 16.5 cm, featuring an exterior Helmholtz coil. The selection of the most suitable Helmholtz coil geometry depends on the specific application, with trade-offs between magnetic field homogeneity and power consumption (53). For example, while circular Helmholtz coils provide superior magnetic field uniformity, they may require more power compared to square configuration. The top and bottom windings are 6 cm apart, with each winding layer occupying 1.75 cm. Remington PN155 wires (polyurethane-coated, 155°C) at 22 AWG connect to an external signal generator through openings in the Mu-metal box located at the top surface corners.

Two signal generators are employed in the experiment: the FY6900 DDS waveform generator (Kkmoon), and the 33120A 15 MHz waveform generator (Hewlett-Packard, United States). The E3631A power supply (Hewlett-Packard, United States) is used to provide static fields for the control chamber. The cell-containing flask is positioned at the center of the acrylic box, nestled between the two layers of the Helmholtz coil. The static magnetic field within the incubator is measured using a Walker Scientific FGM-4D2N fluxgate magnetometer (Walker Scientific Inc., United States). The alternating field is measured using a Lutron GU-3001 milli-gauss meter (Lutron Electronics, United States).

### 4.2 Biological materials

#### 4.2.1 HT-1080 fibrosarcoma

The primary experimental focus is on the HT-1080 human fibrosarcoma cell line (ATCC CCL-121). These cells were cultivated in Eagle’s Minimum Essential Medium (EMEM; ATCC 30–2003) supplemented with 10% fetal bovine serum (FBS; ATCC 30–2020). To facilitate cell proliferation, tissue-cultured (TC) treated Corning® 25 cm^2^ Rectangular Canted Neck Cell Culture Flasks with Vent Cap (Corning Inc., NY, United States) were employed. Once cell confluence reached between 70 and 90%, the cells were transferred to new 25 cm^2^ flasks for the initial experiment or for future culturing. The seeding number density for fibrosarcoma is 5,000 cells/cm^2^ for each flask.

To counteract potential issues arising from extended culture durations (more than 4 days), including nutrient depletion and metabolic waste accumulation, growth medium changes were carried out for both control and treated cells on the second and third days after the experiment’s commencement.

Prepared EMEM with 10% FBS was stored at 4°C for preservation and gradually heated to 37°C in a temperature-controlled incubator (set at 37°C) before being used for medium changes. The 10% FBS, when not in use, was kept at −20°C and similarly allowed to reach a metabolic temperature of 37°C before being mixed with EMEM.

#### 4.2.2 Human dermal fibroblast

Human dermal fibroblast (ATCC PCS-201-012) cells were grown on a fibroblast growth medium (Sigma Aldrich 116–500). The cells were cultured in TC Corning® treated flasks of 25 cm^2^ to expand cell numbers. After reaching a confluence between 70 and 90%, the cells were seeded in 25 cm^2^ flasks with the seeding density of 10,000 cells/cm^2^ for the experiment.

### 4.3 Cell growth measurement

In our cell growth experiments, HT-1080 fibrosarcoma cells were cultivated in Corning cell culture flasks with a surface area of 25 cm^2^. To maintain genomic stability, we used cell culture passages ranging from 6 to 12. For the cell counting assay, cells were seeded at a concentration of 125,000 cells per flask and incubated in a 6% CO_2_ environment at 37°C. The cells underwent exponential proliferation under the influence of externally applied magnetic fields and were extracted from the incubator at the end of the fourth day (Figure 6D).

Upon removal of the culture medium, the cells were rinsed with Dulbecco’s Phosphate-Buffered Saline (D-PBS; ATCC 30–2,200) at a volume of 5 mL per flask. After discarding the D-PBS, 1.5 mL of trypsin (ATCC PCS-999-004) was added per flask and the flasks/cells were returned to the incubator for 5 min. Cell detachment from the flask bottom was confirmed under a microscope. Neutralization of trypsin was achieved by adding 4.5 mL of reinforced EMEM medium for HT-1080 fibrosarcoma cells or 1.5 mL of Trypsin neutralizing solution (ATCC PCS-999-004) and 3 mL of fibroblast growth medium for fibroblast cells, per flask. The solution was then transferred to a centrifuge tube and centrifuged at 1500 rpm for 5 min using the Centrifuge XC-2000 Premiere (C & A Scientific, Manassas, VA, United States). Subsequently, the supernatant was removed, and 1 mL of culture medium was added gently, mixed with a pipette to obtain a uniform cell suspension.

A 100 μL aliquot of the cell suspension and 100 μL of trypan blue were mixed gently, with 10 μL used for each individual count. The mixed solution was transferred to corresponding cell slides, which were then randomized and the exposure condition label was temporarily removed before the data were collected to single-blind the result. We employed the Countess II Automated Cell Counter (Thermo Fisher Scientific, Waltham, MA, United States) to determine the cell count upon completing the experiments, ensuring precise measurements of the cell population.

### 4.4 Fluorescence measurements

Fluorescent assay measurements were conducted using an iBidi 24-well plate, with a surface area of 1.54 cm^2^ per well. In each experiment, nine wells were utilized: six wells for cells and three wells for blank subtraction. The middle six wells on two plates, one for the control and one for the treated samples, were seeded at a concentration of 5,000 cells/cm^2^ and incubated at 37°C with 5% CO_2_. A volume of 1 mL of the cell suspension was added to each well to facilitate cell growth and exposure. The selection of six wells located in the middle of each plate was specifically made to measure fluorescence intensities. This choice was made considering the homogeneity of MFs in this region, ensuring consistent measurements across all samples. Within each well, two samples were collected for fluorescence analysis. For blank subtraction, three wells were designated and filled with phosphate-buffered saline (PBS) only. These blank wells accounted for background fluorescence and enabled accurate measurements. Fluorescence studies were performed using a multi-detection microplate reader, specifically the Varioscan LUX (Thermo Fisher Scientific, MA, United States), after 4 days of continuous exposure to the low frequency weak MF superimposed on SMF. Each experimental condition was examined separately, and a total of 6 measurements were taken per parameter. The mean and standard deviation were calculated based on these 6 measurements for each experimental condition.

### 4.5 Membrane potential

Twelve μL of FluoVolt (Thermo Fisher Scientific, MA, United States) dye, 1,000 × and 120 μL 100 × PowerLoad concentrate, and 12 mL PBS were added into a 15-mL tube and mixed to prepare fresh FluoVolt Loading Solution. Medium from adherent cells was removed and cells were washed twice in PBS. One microliter of FluoVolt Loading Solution was added to each well containing cells, the cells were incubated at room temperature for 30 min before fluorescence measurements were taken, and the cells were washed twice and suspended in PBS. The fluorescence was measured using the florescence plate reader with appropriate wavelength settings (excitation at 522 nm, emission at 535 nm).

### 4.6 Mitochondrial calcium

The Rhod-2 AM (Thermo Fisher Scientific, MA, United States) probe was used to determine mitochondrial Ca^2+^ level in HT-1080 human fibrosarcoma cells. Cells were washed and suspended in PBS containing Rhod2-AM (1 μM). Cells were normally incubated with the AM ester for 30 min at 37°C. Before fluorescence measurements were taken, the cells were washed and suspended in PBS. The fluorescence intensity from these cells was measured using the fluorescence plate reader with appropriate wavelength settings (excitation at 550 nm, emission at 580 nm).

### 4.7 Mitochondrial superoxide

We used a fluorescent probe (MitoSOX Red reagent; Thermo Fisher Scientific, MA, United States) that specifically detects superoxide within the mitochondrial matrix. The contents (50 mg) of one vial of MitoSOX mitochondrial superoxide indicator were diluted in 13 μL of DMSO to make a 5 mM MitoSOX reagent solution. The 5 mM MitoSOX reagent solution was diluted in PBS to make a 5 μM MitoSOX reagent working solution. Cells were then washed and suspended in PBS containing the 5 μM MitoSOX reagent working solution. Afterward, cells were incubated for 10 min at 37°C. The fluorescence intensity from these cells was measured using the fluorescence plate reader with appropriate wavelength settings (excitation at 510 nm, emission at 580 nm).

### 4.8 Confluence counting

Cells are seeded in CELLTREAT 48-well tissue culture plate (CELLTREAT Scientific Product, MA, United States) and Falcon® 96-well black flat bottom TC-treated microplate (Corning Inc., NY, United States). Only the center 24 and 60 wells are seeded with the appropriate starting density for the 48 and 96-well, respectively. Confluence data are obtained using a Celigo™ Image Cytometer (Revvity, MA, United States) at the 24-, 48-, 72-, and 96-h mark after first seeding.

### 4.9 Statistical analysis

In each experiment, we collected eight samples, resulting in a total of 16 samples. For each experimental condition, we calculated the mean and standard deviation based on the 16 measurements. For the statistical analysis in this research, MATLAB R2024a (MathWorks, United States) was utilized due to its robust computational capabilities and extensive array of statistical functions. The Two-sample t-test function was used to find significance between treated and control samples with 5% significance level. The star notations (*) for *p* < 0.05, (**) for *p* < 0.01, and (***) for *p* < 0.001 represent significant differences. For generating the plots and visual representations of the data, PRISM 10 (GraphPad Software, United States) was employed, known for its high-quality graphing options.

For confluence data analysis, daily confluence values are modeled using an exponential function, N = N₀eᵃᵗ, where N is the confluence count, a is the growth rate, and t is time. An average initial confluence N₀ is estimated from day 1 data. Each dataset is then normalized by dividing by its mean confluence and multiplying by the average initial confluence. The normalized data are analyzed using an unpaired two-tailed Student’s t-test to compare control and treated groups for each day and for inter-day differences.

## Data Availability

The original contributions presented in the study are included in the article/Supplementary material, further inquiries can be directed to the corresponding author.
