# Supplementary material for: The proliferation rates of HT-1080 human fibrosarcoma cells can be accelerated or inhibited by weak static and extremely low frequency magnetic fields
Source: Front Public Health. 2025 Jun 12;13:1535155. doi: 10.3389/fpubh.2025.1535155 (PMC12199451; doi:10.3389/fpubh.2025.1535155)
Supplement: Supplementary file 1 [file Supplementary_file_1.DOCX]

Supplementary Material

# Supplementary Figures


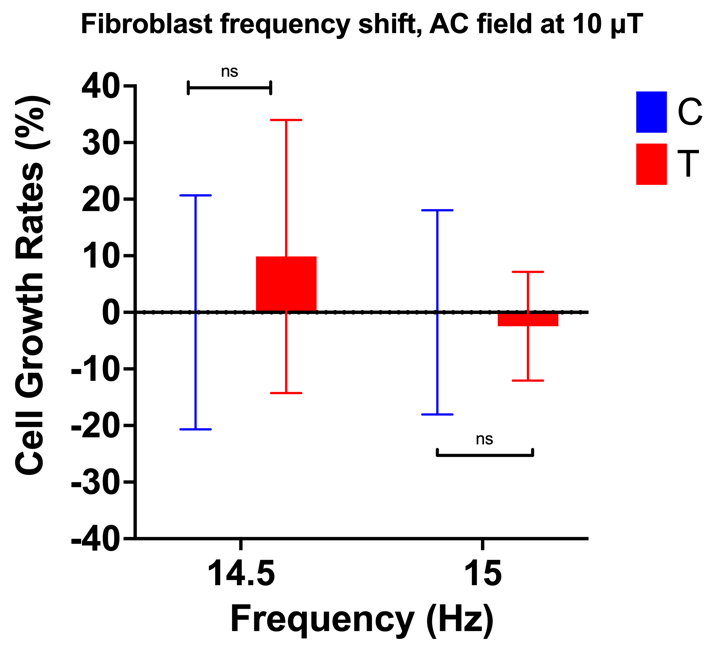


**Supplementary Figure 1.** The cell growth rate of treated (T) and control (C) fibroblasts cells as a function of frequency after a four-day exposure. Normalized mean values with +/- SD of treated and untreated samples (n=16, N=1) are presented. AC sin-wave exposure, DC field = 45 µT.

**(A)
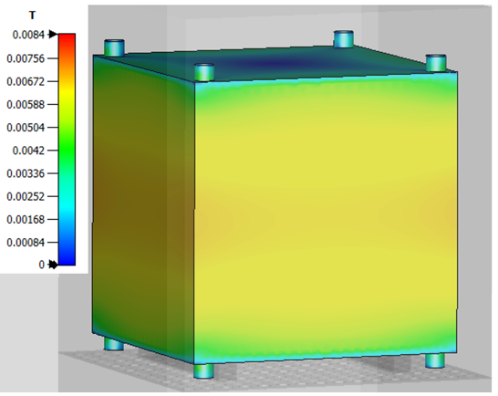
 (B)**
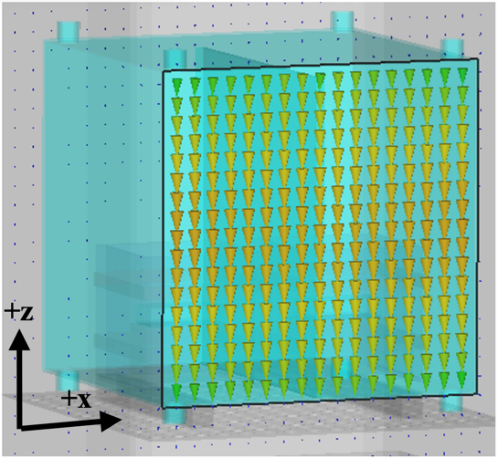


**(C)**
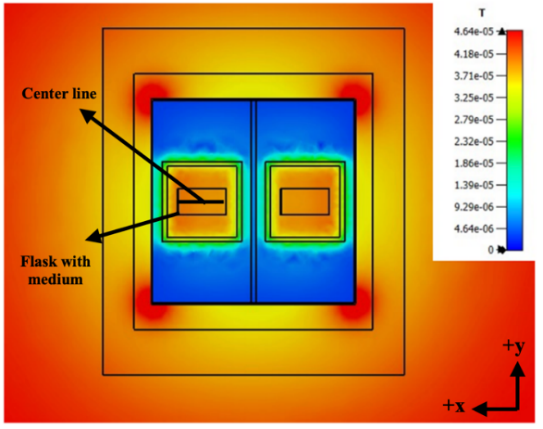


**(D)
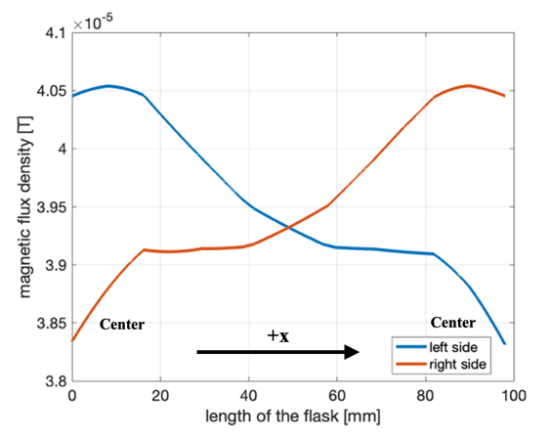
 (E)**
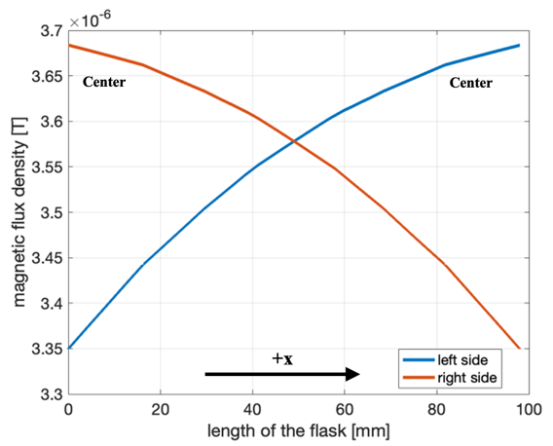


Supplementary Figure 2. Magnetostatic Solver for this evaluation was used with accuracy of 10^-6^ and 862,684 tetrahedrons. (A) The magnetic flux density at the surface of the Mu-metal box. (B) The cross section of the wall of the Mu-metal box with magnetic flux density spread represented by cones. (C) Magnetic flux density spread along the z axis in the cross section corresponding to the middle of the flask. +x-direction is from the leftmost side to the rightmost, and +y-direction is from the back to the front of the incubator. (D) B-field evaluation on the curve along the center line middle of the medium, when both the coils are supplied by DC current of 0.11 A. (E) B-field evaluation on the curve inside the along the middle of medium, when both coils are not supplied by DC current.^[[1]](#footnote-1)^

1. Bajtos, M., Radil, R., Janoušek, L., & Dang, N. (2023, September). Numerical simulations of static magnetic fields with Mu-metal cage shielding. In 2023 24th International Conference on Computational Problems of Electrical Engineering (CPEE) (pp. 1-4). IEEE. [↑](#footnote-ref-1)
